# A Rational Treatment of Prostatic Obstruction in Old Men

**Published:** 1894-07

**Authors:** G. Wiley Broome

**Affiliations:** St. Louis, Mo.


A RATIONAL TREATMENT OF PROSTATIC OBSTRUCTION IN OLD MEN. By G. Wiley Broome, M.D., St. Louis, Mo.—Dr. Broome takes issue, in this paper, with Magill and others, with reference to the advisability of prostatectomy. He also opposes systematic catheterism. The systematic use of the catheter invariably gives rise to infection, and so increases the patient's sufferings. Besides, the systematic use of the catheter soon destroys the ability of the bladder to empty itself, and furnishes an efficient means of evacuating the bladder only for a short time, it being impossible to use the catheter in such a way as to completely drain the bladder. Sir Henry Thompson's assertion that habitual catheterism for two years will permanently destroy the power of the bladder to empty itself, is abundantly confirmed by the experience of many surgeons. Dr. Broome's method is based upon the suggestion of Sir Henry Thompson, which aims to relieve these cases by suprapubic drainage. Dr. Broome's principal improvements consist of opening the bladder at a second operation, by means of puncture with a trocar, and inserting a catheter made self-retaining by means of a screw thread cut upon it. He claims that in his cases of atrophy of the inflamed prostate, the restoration of the function of the urethra was secured by a few months' rest of the bladder secured in this way.—*Modern Medicine, from the Omaha Clinic.*

